# Formation of an electrical coupling between differentiating cardiomyocytes

**DOI:** 10.1038/s41598-020-64581-5

**Published:** 2020-05-08

**Authors:** M. M. Slotvitsky, V. A. Tsvelaya, A. D. Podgurskaya, K. I. Agladze

**Affiliations:** 1 Moscow Institute of Physics and Technology, Dolgoprudny, Moscow Region 141700 Russian Federation; 2 M.F. Vladimirsky Moscow Regional Clinical Research Institute, Moscow, 129110 Russian Federation

**Keywords:** Stem-cell differentiation, Heart development, Heart development, Biological physics

## Abstract

Human induced pluripotent stem cell–derived cardiomyocytes (hiPSC-CMs) serve as an indispensable platform for the study of human cardiovascular disease is human induced pluripotent stem cell–derived cardiomyocytes (hiPSC-CMs). While the possibility of reproducing rare pathologies, patient-specific selection of drugs, and other issues concerning single cardiomyocytes have been well studied, little attention has been paid to the properties of the whole syncytium of CMs, in which both the functionality of individual cells and the distribution of electrophysiological connections between them are essential. The aim of this work is to directly study the ability of hiPSC-CMs to form a functional syncytium that can stably conduct an excitation wave. For that purpose, syncytium forming hiPSC-CMs were harvested and seeded (transferred) on a new substrate on different days of differentiation. The excitation conduction in a sample was characterized by the stability of the wavefront using optical mapping data. We found that the cells transferred before the 20th day of differentiation were able to organize a functional syncytium capable of further development and stable excitation conduction at high stimulation frequencies, while the cells transferred after 20 days did not form a homogeneous syncytium, and multiple instabilities of the propagating wavefront were observed with the possibility of reentry formation.

## Introduction

With the discovery of induced pluripotent stem cells (iPSCs) made by Shinya Yamanaka in 2006^1^ and the development of protocols for the directed differentiation of iPSCs into ventricular CMs^2^, it became possible to generate cardiomyocytes (CMs) in sufficient quantities for laboratory studies that reproduce certain properties of *in vivo* CMs (excitability, contractility, etc.)^3–6^. Cell layers obtained from iPSCs are used in studies of cardiotoxicity and, in particular, in studies of the effects of pharmaceuticals on cardiac electrophysiology (e.g., human ether-à-go-go-related gene [hERG] testing recommended by the Food and Drug Administration [FDA]). One of the basic functions of the myocardium—both as a tissue and in terms of its physiological role—is the transmission of an electrical stimulus through the ventricular tissue, coupled with the mechanical contraction of the ventricle wall^7^. The most common type of ventricular arrhythmia, fibrillation, is associated with a loss of the stability of the electrical stimulus. The synchronous and stable conduction of an impulse in the form of a propagating wave of excitation sets the regularity and direction of mechanical contractions of the ventricular tissue, while a change in the wave regime leads to the inconsistent contraction and improper pumping of blood, and, consequently, to the inefficient performance of the entire cardiovascular system. The presence of functional heterogeneities in the myocardium plays a decisive role in disorganization of the conduction of the excitation wave through the tissue; it is in the vicinity of such heterogeneities that reentry waves may occur, which are the fundamental cause of a transition from a normal conduction regime to a chaotic one, manifested as a ventricular fibrillation^7,8^. In practice, the occurrence of reentry is an unpredictable and practically irreversible process; as such, according to World Health Organization (WHO), cardiovascular disease (CVD) is the most common cause of death among the adult working population in developed countries^9,10^.

Generally, when creating iPSC-based tissue-engineering structures used to study reentry, the main emphasis is on the electrophysiological maturity and purity of the cultivated CM population, which are components of the conductive tissue, and the task of tracking the development of intercellular connections and pathways remains unfulfilled. One of the features of iPSC-CM is the fetal-like phenotype, which causes cells not have a clear structure or shape^11,12^. Consequently, modern three-dimensional printing methods do not allow the manipulation of the location of cell contacts or cell orientation^13,14^. In cultured CM layers, the possibility of a reentry is shown, the cause of which may be broken cell connections due to the separation of CMs by fibroblasts or the uncoupling of gap junctions, leading to the possibility of a unidirectional block and the reentry origination^6,14–17^.

Several teams working with various iPSC lines showed the existence of a correlation between the stability of excitation conduction in tissue and the properties of CMs that change with the stage of differentiation^11,12,18–22^. The presence of a temporary perinatal window was detected in the early stages of differentiation, when the cells are most susceptible to changes under the influence of external factors and to the formation of intercellular contacts^18,22^.

This article is devoted to reviewing the ability of CMs to form a new conductive syncytium at different stages of differentiation and to study of its properties. For this purpose, optical mapping of samples of a layer of cardiac tissue with dimensions greater than the excitation wavelength and capable of supporting one or several reentries at different frequencies of the electrode stimulus was used^15,16^. The measure of the functional homogeneity of the sample was based on the excitation wave pattern and its changes at higher stimulation frequencies.

## Materials and methods

### Cells lines and differentiation protocols

In this paper we used m34Sk3 and iSMA6L cell lines reprogramed from a healthy donor^15,16,23^. Necessary information on cell lines is given in the studies^15,16^. Direct cardiac differentiation was performed in 24-well plates according to modification of the Gi-Wi protocol^2^, given in the study^16^.

A more complete immunofluorescent analysis and staining protocols are presented in previous works^15,16^. Information on the differentiation efficiency of the m34Sk3 cell line can be found in the article^23^.

### Cell transfer procedure

Cardiomyocytes were disaggregated using TrypLE Express (1X) (https://www.thermofisher.com/order/catalog/product/12605036) to a unicellular state according to the following protocols^24^.

Cells are incubated for 5 minutes (instead of 10 minutes to minimize cell damage) in 700 ml of a disaggregating solution at physiological temperature in a CO_2_ incubator.

A cell suspension (usually all cells are torn off the substrate, but small clusters of cells are further split mechanically by the fluid flow) is washed off from the original substrate with RPMI 1640 medium with 1 × B27 Supplement with 10 μM Y27632 (StemRD, USA). The suspension is centrifuged for 5 minutes at 200 g, all cells are precipitated. Then the supernatant (containing the medium and the disaggregating solution) is completely drained, the cell suspension is again mixed in RPMI 1640 medium with 1 × B27 Supplement with 10 μM Y27632, after which all cells are plated on a new substrate. The new substrate was coated with Matrigel according to the same protocol as for iPSC transplantation for cell differentiation^16,23^. In this work, we performed cell transfer procedure on different days of differentiation. In the description of the results, a conditional division into two groups is sometimes used: transferred before day 20 and transferred after day 20.

### Analysis of the consistency of calcium dynamics in the sample (correlation)

Processing of optical mapping recordings was carried out in the ImageJ program. Calculations were performed in Python 3 (Jupyter Notebook) using numpy library. Excitation recordungs are made by fluorescence of calcium-dependent dye Fluo4-AM. The operation of the algorithm is based on the following assumption: if the sample of the excitable medium is homogeneous, then the wave will propagate in all directions isotropically. So, the wave propagation speed will be constant and determined by the properties of this excitable medium (in particular, the hiPSC-CM monolayer).

On the wave propagation path, two sites of equal area are chosen. In the case of optical mapping with calcium dyes, the fluorescence peaks can be regarded as cell excitation and local minima as a resting state.

During recording of the optical signal, electrode stimulation is switched on at a constant frequency (60 bpm or 100 bpm), leading to an excitation wave propagating through the sample. Intensity records from both sites are superimposed at the first recorded peaks corresponding to the passage of the first wavefront (see the lower graphs in Fig. 3A–F).

**Figure 1:**
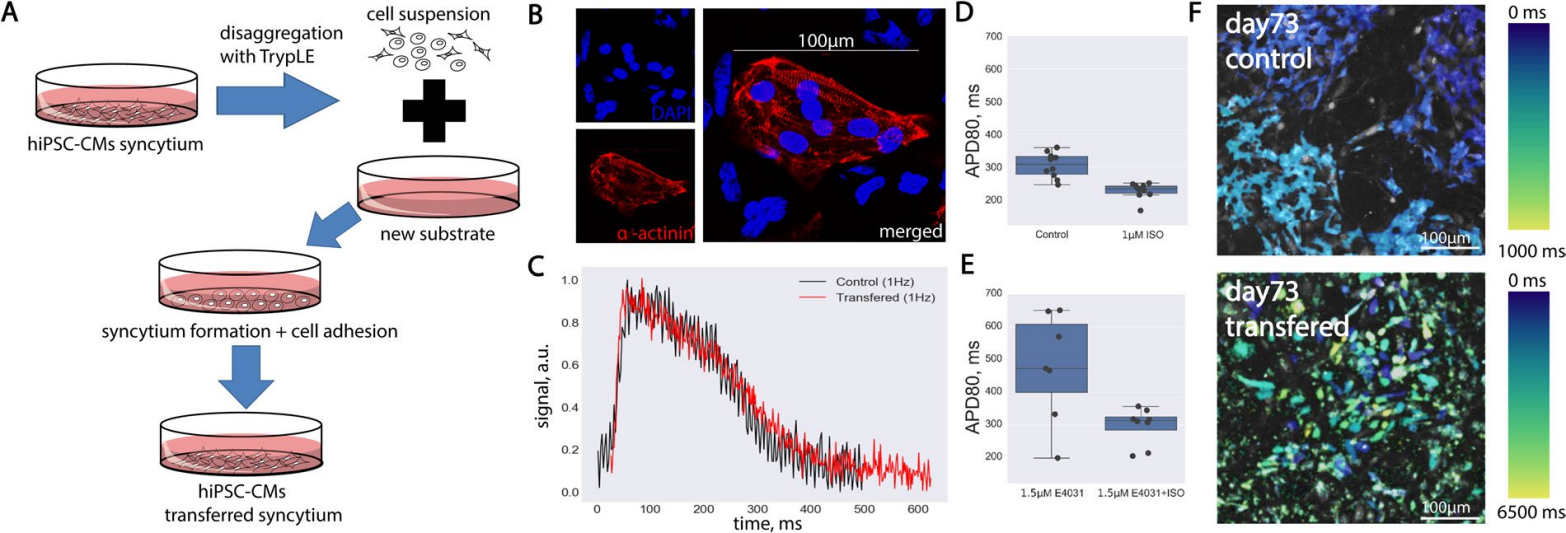
hiPSC-CMs transfer experiment. **(A)** Design of the experiment, transfer procedure. (**B)** Immunocytochemistry of single cardiomyocytes after disaggregation. (**C)** Optical recording of the action potential of transferred cardiomyocytes with frequency of 130 Hz. The black curve shows the control action potential. Red is the action potential of cells after transfer. The numerical changes in APD80 under the action of isoprenaline and E4031 are shown in Figures (**D**,**E)**, respectively (n = 3 is the number of different tissue samples). (**F)** shows activation maps of the hiPSC-CMs syncytium excitation wave for the control sample (above) and the sample obtained by transfer (below).

**Figure 2:**
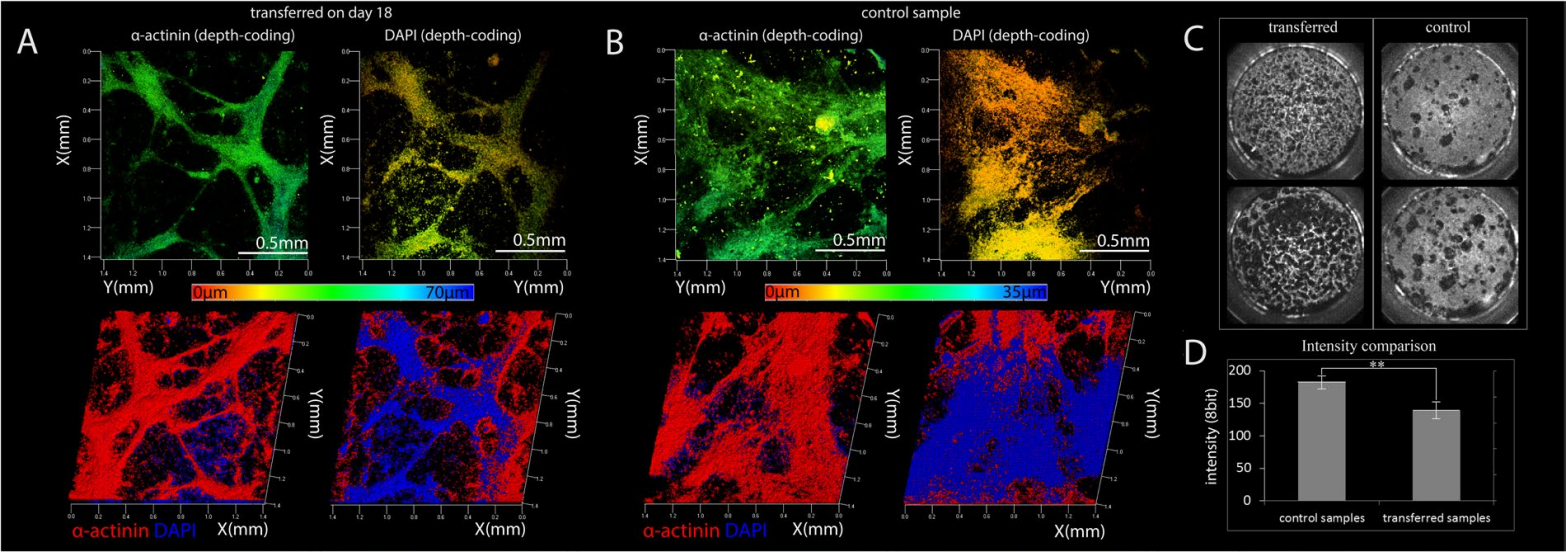
Three-dimensional reconstruction of the structure of the cardiac layer, compiled from confocal images (20 confocal slices). (**A**) Sample received by transplant (transferred on 18th day, stained on 50th day). The top pictures display DAPI and alpha-actinin separately in depth-coding, showing the exact coordinate along the vertical axis. The lower figures show a qualitative picture of the relative positions of these two markers. Figure (**B)**. Presents the structure of the control sample (stained on 50^th^ day). (**C)** Comparison of the structure and distribution of conductive cells of control samples and transferred ones (amplitude charts, sample size - 15 mm). A qualitative comparison is presented in the upper figures, where black is the non-conductive zone of the sample and white is the conductive zone. The color is determined in accordance with the maximum amplitude of the intensity fluctuations in the dependence of the optical mapping experiment. A numerical comparison of data is presented in the diagram (**D)**. **p-value <0.001 (n = 10, m = 11).

**Figure 3:**
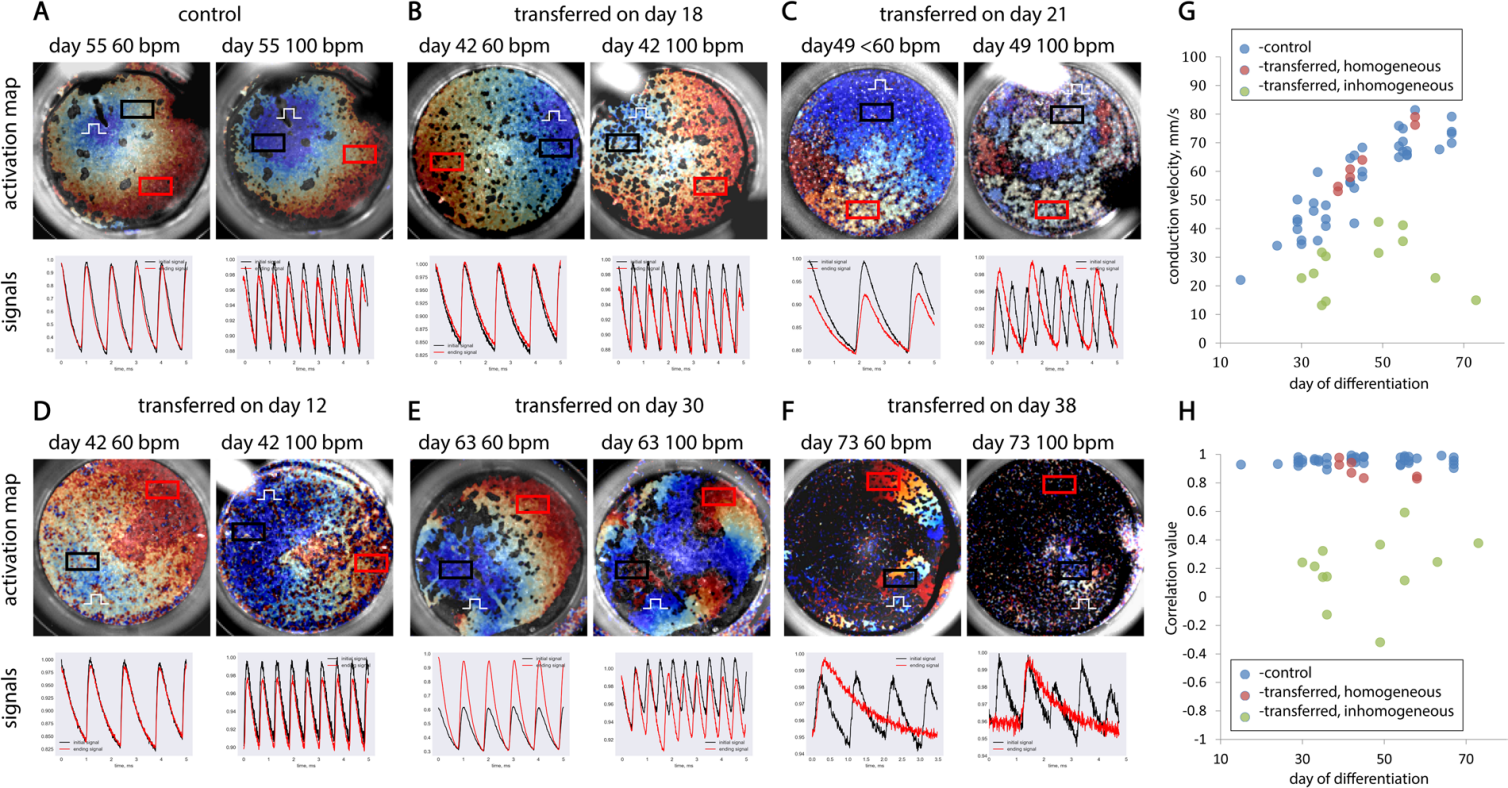
Optical mapping of samples at a stimulation frequency of 60bpm and 100bpm. Figures (**A–F)** show the activation maps (sample size - 15 mm) for the calcium wave recorded during optical mapping for 2 stimulation frequencies: 60bpm (or less) and 100bpm. Under each map records of the intensity of the calcium signal in two different areas are given. (**G**) Graph of wave conduction velocities measured on different days of differentiation. (**H)** Values of the correlation for each sample. On both graphs, blue dots represent control samples, and red and green - transferred ones.

If the assumption of homogeneity and isotropy of the sample is correct, then the correlation of signals after alignment will tend to unity. This corresponds to the case when the ups and downs of the concentration of intracellular calcium at one end of the monolayer at regular intervals lead to its ups and downs at the opposite end of the sample with the same frequency. Otherwise, if the wavefront is broken at inhomogeneities, this will lead to a loss of consistency and, consequently, a decrease in the correlation of signals.

### Optical mapping

Before the optical mapping procedure, all chemical solutions were warmed to 37 °C. The optical mapping with Fluo4-AM was carried out according to the protocol described in^6^. The calcium waves were visualized and recorded by a high-speed imaging setup comprising an Olympus MVX-10 Macro-View fluorescent microscope and a high-speed Andor EM-CCD Camera 897-U. The potential-sensitive dye FluoVolt (https://www.thermofisher.com/order/catalog/product/F10488?SID=srch-srp-F10488#/F10488?SID=srch-srp-F10488) was used to record the action potential. The dye was dissolved in a tyrode according to the protocol (https://assets.thermofisher.com/TFS-Assets/LSG/manuals/MAN0009668_FluoVolt_Membrane_Potential_Kit_UG.pdf).

The optical mapping was done in less than 30 minutes so as not to damage the cells. After the optical mapping, the sample was washed a couple of times with PBS solution. The Tyrode solution was exchanged with RPMI 1640 medium with B27 supplement. The sample was placed back into the incubator for later experiments.

The speed of the excitation wave in each case is determined by the spatio-temporal scan, so equipment errors may be calculated using the angle-measure error as a dominant effect. Activation maps were constructed by applying cycles of the minimal intensity subtracting followed by a Kalman filter with an acquisition noise variance estimate of 0.05 and a bias of 0.80 placed on the prediction for the initial video.

### Data processing

All videos from optical mapping were processed in the ImageJ program. The activation and amplitude maps were built using the Wolfram Mathematica 9 program and Image J. The image processing from the confocal microscope was performed in the Zen program corresponding to the microscope software.

Statistical significance of differences between groups determined using ANOVA followed by Fisher’s least significant difference test for group comparison; the differences were considered significant at p < 0.05.

The Principle Components Analysis (PCA) and box-counting algorithm were performed in Jupiter notebook using Python 3 sklearn, pylab and numpy libraries. The number of principal components was chosen in such a way as to maintain 90% dispersion. Data preprocessing and normalization were provided in Microsoft Excel.

### Ethical approval

The cell line m34Sk3 is provided by the E. Meshalkin Novosibirsk Scientific Research Institute of Circulation Pathology and handling approved by the Institute of Circulation Pathology Ethics Committee (#27, March 21, 2013). The generation of iPSC line from cells donated by patient with informed consent described in^16,23^. All experiments and procedures were performed in accordance with principles for human experimentation as defined in the 1964 Declaration of Helsinki and its later amendments and were approved by the Scientific Council of the MIPT Life Science Center.

All applicable international, national, and/or institutional guidelines for the care and use of cell lines were followed.

## Results

### Cell transfer

Starting on the 12th day of directed differentiation, consistent coordinated contractions of individual tissue sections appeared in the samples, and it became possible to register the excitation wave in the optical mapping experiments. According to the chosen differentiation protocol, further development of the samples occurred under constant conditions: the composition of the components of the culture medium remained unchanged, starting from the 8th day of differentiation. The occurrence of contractile activity and the constancy of the culturing conditions allowed us to consider the cells to be ready for transfer and the subsequent restoration of the cellular syncytium on a similar substrate and in a similar culture medium.

The CM transfer process was the same for all samples. The transfer plan is shown in Fig. 1A, while a detailed description available in the “Methods and Materials” section of this paper. All samples used in the study were transferred no more than one time within the time interval between 12 and 38 days of differentiation. Thus, the samples were divided into two groups: the control group—that is, the samples formed naturally in the process of differentiation and not transferred—and the transferred group—that is, samples formed after the relocation of the cells to a new substrate.

The phenotype and electrophysiological properties of the cells after transfer were checked using immunocytochemistry and optical mapping (Fig. 1B–E). A comparison of the action potential of the transferred and control CMs of the m34sk3 line is shown in Fig. 1C, and in both cases, the APD80 value reached approximately 350 ms.

Special attention was paid to testing the potassium channels responsible for slow delayed rectifier potassium current (IKs) and rapid delayed rectifier potassium current (IKr), as the ability of potassium channel proteins to vesiculate in the cell cytoplasm after cell disaggregation is known^25,26^. Activity was tested using E4031 (IKr blocker) and isoprenaline (IKs activator). The corresponding increase in APD80 with the addition of 1.5 μm E4031 and the shortening with the addition of 1 μm isoprenaline are shown in Fig. 1E,D, respectively. The formation of the action potential and its sensitivity to E4031 (as an IKr blocker) and isoprenaline (as an IKs activator) indicates that the selected isolation method does not impair the functioning of potassium channels (Fig. 1C–E).

The integrity of the structures of the cytoskeleton (F-actin protein) and of the contractile apparatus (alpha-actinin) were confirmed by the immunocytochemistry of isolated transferred CMs (Fig. 1B).

Despite the similarities between the transferred and non-transferred cells, optical mapping experiments revealed differences in the samples in conducting an excitation wave between cells. Figure 1F shows the activation chart of the control sample and transferred sample. The activation chart of the transferred sample (lower figure) is characterized by the non-synchronous excitation of neighboring cells, which makes the picture different from that of the sample that did not undergo disaggregation and transfer (upper figure, synchronous excitation of all cells).

In order to understand the difference between wave patterns in transferred and non-transferred samples, we compared their structures in greater detail. Because the efficiency of differentiation of iPSCs into CMs for the m34sk3 line is about 50% on the 20th day of differentiation^23^, it is necessary to monitor the relative position of CMs and non-conductive products of differentiation in the syncytium. Using immunocytochemistry and confocal microscopy, it is possible to reconstruct the bulk structure of a syncytium. The protein-specific markers 4′,6-diamidino-2-phenylindole (DAPI) and alpha-actinin were selected for the contractile apparatus of cardiac cells. Using a confocal microscope, a series of 20 slices was taken with a step of 3 μm for a 1.5 mm sample fragment. The spatial arrangement of the cells can be identified by the locations of their nuclei, and the presence of alpha-actin can indicate cardiac cells (Fig. 2A,B). A more detailed assessment of their relative positioning is presented on maps with depth-coding (Fig. 2A,B, upper figures). Both the control and transferred samples are characterized by the fact that the thickness of the syncytium can be two to three cell layers. In both cases, CMs are located mainly on the surface of the syncytium.

The assumption of the assemblage of CMs on the surface of cell layer makes it possible to estimate the population of functional CMs in the resulting syncytium based on optical mapping experiments. Using an optical signal recorded during the propagation of the excitation wave in a monolayer with calcium-sensitive fluorescent dye, a chart can be constructed in which the brightness of a pixel is determined by the amplitude of the signal from this pixel during recording. So, pixels with an intensity fluctuating around zero will be black in color, while pixels the with highest recorded fluorescence intensity will appear white. The presence of noise and limited spatial resolution (approximately 0.2 megapixels per 1 million cells) in the recording lead to all pixels being shaded in gray, but with pixels indicating greater signal amplitude being brighter grey; this indicated, accordingly, that the CMs in the vicinity of such pixels show greater density. The effect of noise was minimized by averaging the intensity over the entire area of the sample. The average intensity of the amplitude charts for all control samples was in a rather narrow range, which allowed us to judge that all control monolayers were composed of approximately the same number of functional conducting myocytes. The control samples were characterized by a uniform distribution of CMs over the entire area with localized non-conducting regions formed, apparently, by clusters of non-conducting cells.

However, while the average intensity values for the transferred samples also coincided, they differed from the values seen in the control samples. Similar to the control samples, all the transferred samples had a single characteristic monolayer structure with uniformly distributed CMs forming pathways, but the total number of excitable cells was lower and the non-conducting regions were distributed uniformly over the entire area of the sample (Fig. 2C). Thus, the spatial distribution of the CMs changed after transfer; however, their total number was constant for all the transferred samples, regardless of the day on which the transfer and optical mapping were performed (Fig. 2C,D) (p = 0.0001, n = 10 for transferred samples, m = 11 for control samples). The proportion of CMs in the control samples did not change with time and was about 50% of the total number of cells^23^. Based on the intensity values obtained from the amplitude maps, we studied the efficiency of differentiation into hiPSC-CMs at various differentiation stages for our protocol (Supplementary Fig. 1A,B). A similar approach to monitoring the differentiation efficiency was applied in^22^. The absence of statistically significant differences in intensity on different days of differentiation allows us to judge that the effectiveness of the protocol was unchanged over the entire studied time interval. Relative percentage of atrial, ventricular, or conducting system CMs in hiPSC-CMs was checked for 50th day culture (n = 5) according to APD50 measurements with optical mapping (Supplementary Fig. 1C,D).

### Analysis of optical mapping experiments

Activation maps (Fig. 3A–F) demonstrate the conduction of an excitation wave at different stimulation frequencies. The speed of the excitation wave in each case was determined using a spatio-temporal scan, and the obtained values are indicated by blue dots on the graph in Fig. 3G, in the axes of speed and day of differentiation. In order to provide the maximal information about the excitation wave pattern in numerical form, the correlation between the calcium dynamics at opposite ends of the sample was calculated at a frequency of 100 bpm (hereafter referred to as “the correlation”; Fig. 3H). A detailed algorithm and its justification are given in the “Methods and Materials” section.

For the entire section of control samples at the selected stimulation frequencies (i.e., 60 bpm and 100 bpm), the excitation wave was stable and did not exhibit wave breaks. In turn, the correlation for all control samples (n = 39) showed values ranging from 0.8 to 1.0, and the scatter can be explained by recording noise (Fig. 3H). Activation maps of the control samples show front sphericity and continuity and the absence of points of singularity (see the example in Fig. 3A).

The conduction patterns in the transferred samples turned out to be different; according to the conduction properties, two conditional groups can be distinguished: homogeneous and heterogeneous. Samples that did not exhibit unstable conduction or wave breaks at the selected frequencies (n = 7 out of a total of 19 transferred samples) were classified as homogeneous (see the examples in Fig. 3B,D). The correlation for this subgroup of transferred samples also showed values ranging from 0.8 to 1.0. The values of the conduction velocity and correlation rates for these samples are presented on the graph in Fig. 3H, indicated by red dots. If, in the sample—for at least one of the selected stimulation frequencies—it was observed that there was disorder in the wave conduction and/or a mismatch in the frequency of excitation of the sample with the frequency of the electrode stimulus, then the sample was considered heterogeneous (Fig. C,E,F). Consequently, the heterogeneity of the sample was manifested by the curvature of the wave front on the corresponding activation maps and the appearance of singular points, as well as by a decrease in the correlation value (i.e., to <0.8). The speed and correlation values for these samples are represented by green dots in the graphs in Fig. 3G,H, respectively. Thus, correlation as a method for assessing the functional homogeneity of samples did not show false positive or false negative results in a series of experiments—that is, for all samples with wave breaks, the correlation was lower than 0.8, and for all samples without wave disturbance, it was higher than 0.8. To summarize, the optical mapping of each sample produced a set of numerical descriptor values: the speed of the excitation wave (V), the correlation (C), and the maximum captured frequency of stimulation (N).

**Figure 4:**
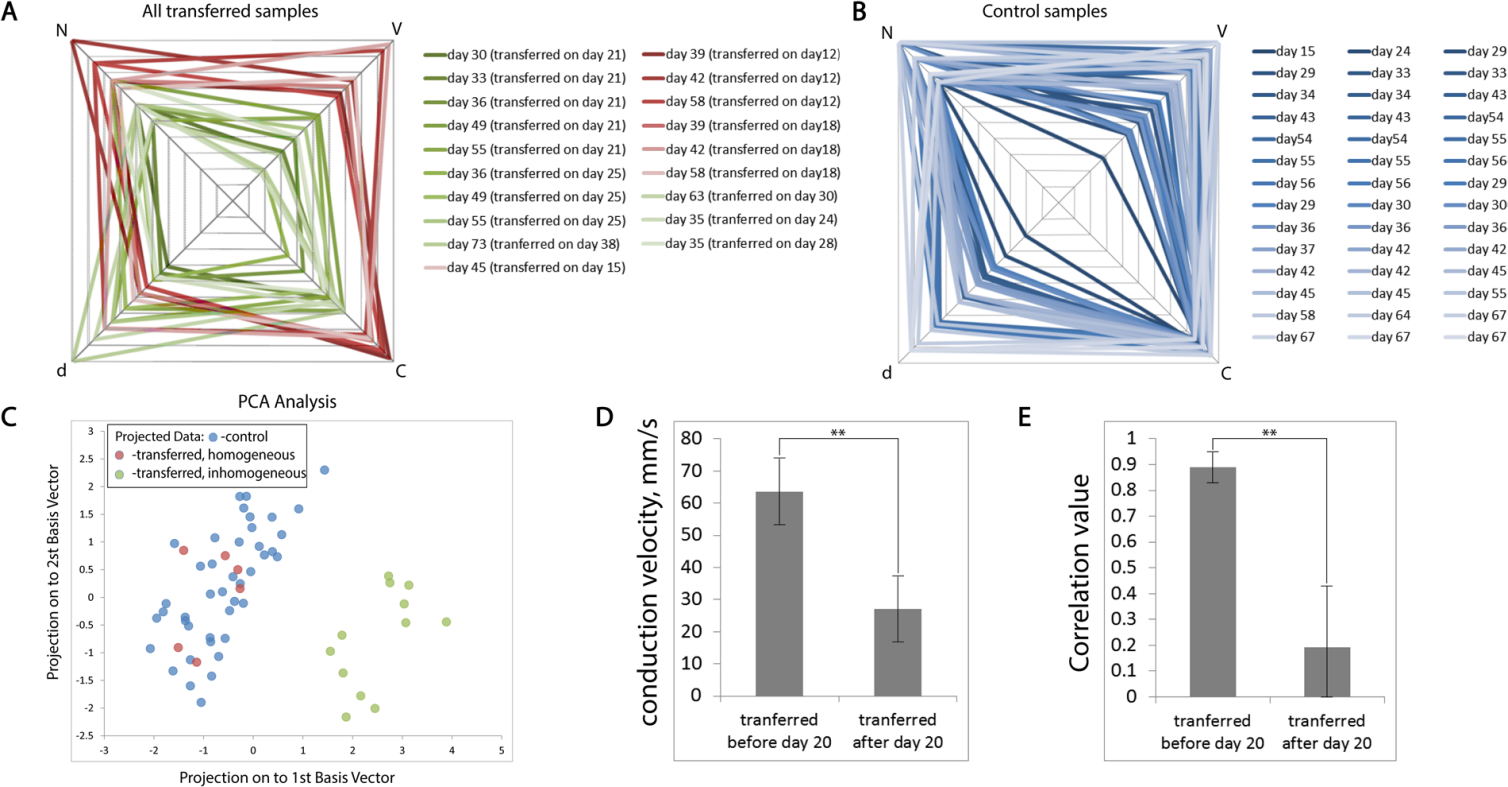
A petal diagram with descriptors for all transferred samples. (**A)** similar diagram for control samples is shown in Figure (**B,C)**. PCA analysis of the entire samples’ data. (**D)** Statistical significance of differences in speed for samples transferred before 20 days or after 20 days of differentiation. (**E)** Statistical significance of differences in speed for samples transferred before 20 days or after 20 days of differentiation. **p-value < 0.001 (n = 7, m = 16).

We next introduced the following assumption: when transferred onto a new substrate, CMs randomly mix with non-conducting cells. Accordingly, the distribution and size of inhomogeneities capable of interrupting the propagation of the excitation wave are random (i.e., independent of any other quantities). As such, each of the selected descriptors from sample to sample can behave as a random variable (Fig. 3G,H). This assumption is our primary hypothesis, and we analyzed the data obtained as a multidimensional random variable. We will next present the entire variety of results obtained on the petal diagrams, along the axes of which the values of the selected descriptors were plotted (i.e., V, C, and N), and the day from the start of differentiation on which the optical mapping of the sample was performed. In this view, each sample is displayed in a quadrangle (Fig. 4A). By analogy with the graphs in Fig. 3G,H, homogeneous samples (i.e., those conducting excitation waves at all frequencies without front discontinuities) are indicated by red tetrahedrons, and heterogeneous samples (i.e., those having conductivity disorder) are indicated by green tetrahedrons. Such a representation is enough to facilitate the estimation of the expectation and variance of the assumed random variable. Moreover, the entire range of values on each axis does not have obvious gaps. A similar representation for control samples is presented in Fig. 4B.

**Figure 5:**
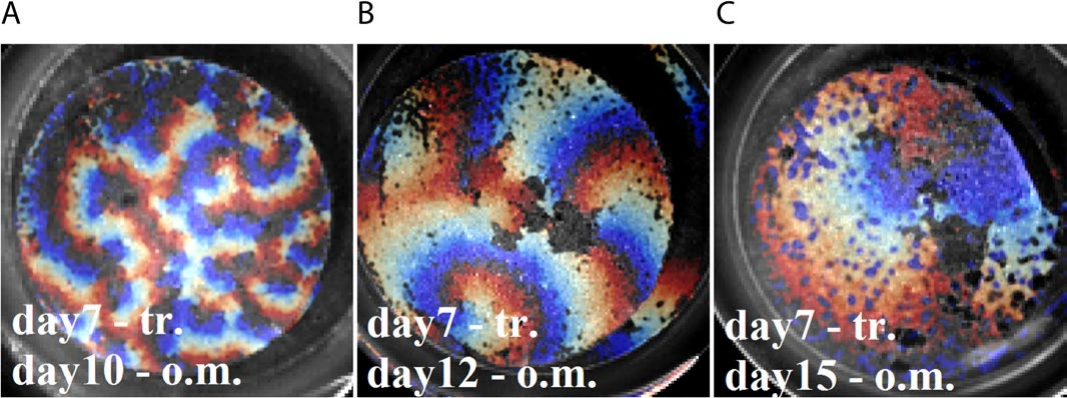
Activation maps, representing the shapes of the wavefronts of excitation on the sample transferred on the 7th day of differentiation. The upper caption in the picture reflects the day of transplantation, and the lower one - the day of differentiation, on which the optical mapping procedure was performed. (**А**) Optical mapping on day 10 (3 days after transfer), (**B**) on day 12 (5 days after transfer) and (**C**) on day 15 (8 days after transfer). Sample size - 15 mm.

It should be noted, however, that the selected descriptors do not necessarily represent values orthogonal to each other. For example, in a previous work^15^, it was shown that the speed of the wave and the captured frequencies are directly dependent on the day of differentiation, which is also one of the selected descriptors. To eliminate linear dependencies in the set of multidimensional data, we used the principal component method, while reducing the dimension of the data to two-dimensional (i.e., choosing the first two principal components) allowed us to obtain a qualitative picture of the distribution of points of our random variable in multidimensional space (Fig. 4C). In the space of the first two main components, a separation of the entire data sample of the transferred samples into two isolated clusters was observed. Homogeneous samples were separated from heterogeneous samples, which allowed us to characterize the random variable under study as bimodal—some of the points were grouped around one expectation (n = 7) and the rest (n = 12) around another. The statistical significance of differences between the two subgroups (i.e., samples transferred before 20 days and after 20 days of differentiation) is presented in the diagrams in Fig. 4D,E (for both diagrams p-value is <0.0001, n = 7, m = 12). It is important to note that the time period between transferring the cells and the optical mapping procedure was no less than 10 days. According to our observations, it takes about week after transfer to restore syncytium functionality (Fig. 5).It is worth noting that the conduction properties (for example, V and C) were recorded on tissue sites having a size of the order of several millimeters. So, wave velocity measurements were carried out on linear sections from one to several millimeters in length, and signal correlation was measured in two sections, separated from each other by 10 mm or more.

Summing up, there are significant differences (p = 0.012526, n = 5 in both groups) in the properties of the excitation wave conduction in samples transferred on different days of differentiation. The statistical significance of these differences is evident when comparing two groups of samples: transferred up to 20 days of differentiation (and mapped on any subsequent day, n = 7), and transferred after 20 days (n = 12).

A similar comparison was made for another line of hiPSC-CMs to confirm that the observed effect was not an artifact arising from the choice of the cell line (m34Sk3 with Gi-Wi protocol). For transferred samples of the iSMA6L line (n = 6), the values of descriptors V, C, N were recorded and compared with the corresponding values for control samples of the same line on the same day from the beginning of differentiation. The values obtained are denoted by dV, dC and dN, respectively, and are shown in the petal diagrams (Supplementary Fig. 2A,B). In the case of the iSMA6L line, the samples transferred before 19 days showed minimal differences in the conduction properties (dV, dC) from the control, reproducing the same effect as was observed on the m34Sk3 line. (Supplementary Fig. 2C,D)

**Figure 6:**
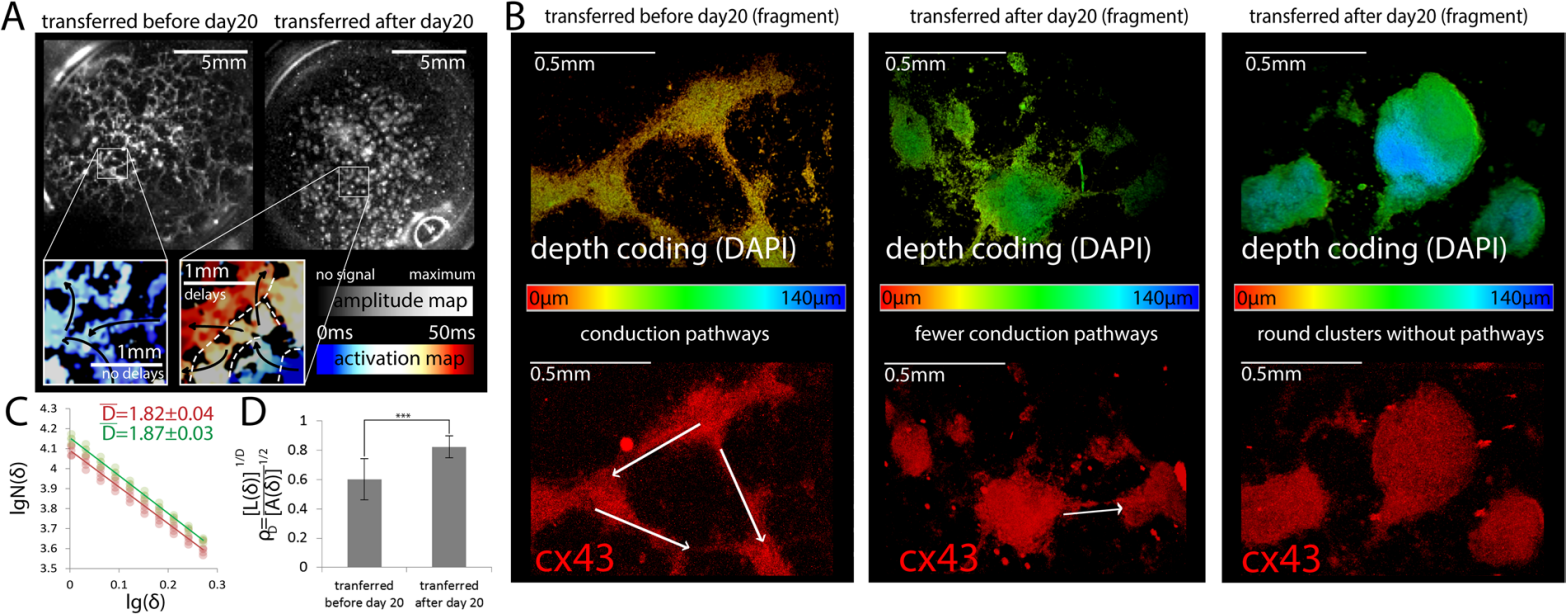
Comparison of the conductivity structure for transferred samples. (**A)** Amplitude and activation maps of transferred samples. The left side shows a typical structure of a sample transferred before the 20th day of differentiation, in the right - the sample transferred after the 20th day of differentiation. In the lower left corner, the activation maps of the excitation wave for the samples are shown. (**B)** Immunocytochemistry of different tissue fragments of transferred samples. The top figure is depth coding DAPI. The bottom figure is cx43. This images show three different typical cases (on the left - the presence of developed conductive pathways between cell clusters, in the middle – minimal presence of conductive pathways, and on the right – absence of any conductive pathways between the clusters). The presence and absence of possible conductive pathways between the clusters is indicated by arrows. (**C)** Determination of the tissues’ fractal dimension of the Hausdorff box-counting method. Red color indicates samples transplanted before 20 days, and green - after. (**D)** Comparison of the ratio of perimeter (L(δ)) and area (A(δ)) of tissue sites between samples transplanted before the 20th day of differentiation and after (δ – standard size). ***p-value < 0.05 (p = 0.012526, n = 5 in both groups).

Amplitude maps allow a more detailed examination of the structure of the conducting pathways formed after tissue transfer, including the comparison of the typical picture of tissue structure for samples transferred before 20 days and after (Fig. 6A). Visually, in samples transferred before 20 days, there are more branched pathways. Whereas in the samples transfer after 20 days, most of the cardiomyocytes are grouped into separate clusters. The transmission of an excitation wave between such clusters can lead to delays in conducting (see activation charts in Fig. 6A below) and, consequently, to a decrease in the speed of conducting an excitation wave.

Figures on panel 6B presents the variety of geometric shapes of cell clusters that we found in immunocytochemical studies of the transferred samples. The left picture shows developed conductive paths with a large cross-section, optimal for the rapid conduction of the excitation wave. The middle picture shows paths that are smaller in size and number, which can complicate the propagation of excitation between clusters. The right picture shows the lack of connections between clusters. In addition to a whole tissue visualization, a high-resolution local visualization of Cx43 presented in supplementary information (Supplementary Fig. 3A,B). Obviously, in the different structures of the monolayer there is a different ratio of the perimeter of the cell cluster to the area occupied by it. In the latter case (round clusters without conducting paths, right picture), the ratio of the perimeter to the area will be the smallest, and in the case of developed conducting paths, it will be the largest. In order to characterize the prevalence of all three cases of hiPSC-CMs throughout the sample, an analysis of fractal measures for activation charts was carried out.To assess how significant and characteristic the differences in the structure of the tissue between the samples were obtained, a method similar to^27,28^ was applied. An assessment of the fractal dimensions of the tissue according to Hausdorff gives the following values (Fig. 6B): D = 1.82 ± 0.04 for samples transferred before 20 days, and D = 1.87 ± 0.03 for samples transferred after 20 days. The differences between the two groups mentioned become significant (p = 0.012526, n = 5 in both groups) for evaluation by the Mandelbrot ratio^29^, which characterizes the ratio of the perimeter and the area of fractals (Fig. 6C). The area and perimeter of the cluster are designated as A and C, respectively, δ is the size of the standard (limited by the size of the pixel)

1 $${\rho }_{D}=\frac{{[L(\delta )]}^{1/D}}{{[A(\delta )]}^{1/2}}$$

## Discussion

The presence of a bimodal data distribution allowed us to propose a new hypothesis to replace the initial assumption of a random variable characterizing the properties of the sample after transfer. The only recorded parameter that reliably predicted which of the clusters the point fell into was the differentiation stage, at which the cell monolayer was directly transferred. As such, all samples recognized as homogeneous were seeded before 20 days of differentiation, while all heterogeneous samples were seeded after 20 days (see the legend in Fig. 4A). Because all other experimental conditions remained unchanged from experiment to experiment, we believe that this difference can indeed be explained by the specific properties of hiPSC-CMs in the early stages of differentiation, which allow for a full recovery of the syncytium functionality of the cells after being transferred.

The border drawn on the twentieth day is relatively conditional. Two characteristic groups can be distinguished from the entire complex of experiments on transplanting samples of the m34Sk3 line. In our data sample, the most recent day of transplantation, which gave a homogeneous monolayer, is day 18; the earliest day of the transplantation, which gave heterogeneous syncytium, is 21 (Fig. 2B,C, respectively). In this case, the following condition is fulfilled: all samples transplanted before 18 days also fell into the first cluster (n = 7), and all samples transplanted after 21 days (n = 12) fell into the second cluster (Fig. 4C). Two possible outcomes are demarcated by a small (in terms of hiPSC-CMs maturation time) time interval between 18 and 21 days. For simplicity, we believe that the desired time interval is in the vicinity of 20 days of differentiation and takes about 3-4 days. This representation of the results was obtained in detail on the m34Sk3 line and is applicable primarily to this line. According to our data, the effect is reproduced on another cell line (iSMA6L, Supplementary Fig. 2).

The point of focusing on the upper time threshold is to determine the narrowest time corridor for further analysis of the expression profile of hiPSC-CMs. The period of maturation of cardiomyocytes in the process of differentiation is about 2-3 months. We are dealing with a switching process that takes only a few days. As a result, CAGE screening in the vicinity of this narrow (relative to the maturation time of hiPSC-CMs) time interval leading to important changes in the properties of hiPSC-CMs will be most effective for obtaining new fundamental data.

The nature of the observed differences in the conduction properties of the samples transferred before and after day 20 are extremely complex and require a detailed and complete analysis of cell expression between 12 and 20 days of differentiation. However, in scientific practice, there is a similar example that sheds light on these differences: It is known that CMs isolated from the cardiac ventricles of neonatal rats are able to form a conductive syncytium only if the rat is no more than five days old. The case of neonatal rat CMs is characterized by the same sharp transition, as after five days, the ability of CMs to form functional connections decreases sharply or almost completely disappears.

The presence of cellular bonds can also be investigated by immunocytochemistry using antibodies of the Сx43 protein, which is the main structural unit of gap junctions. The dominant view is that the electrical coupling between the cells is provided by gap junctions, GJ. This may lead to a conclusion that the observed phenomenon can be explained by differences in the expression of Cx43 protein, which forms GJ. However, it is known that in early cardiomyocytes the level of Cx43 expression is reduced compared to the later phases^30–32^. If we draw an analogy (to a certain extent) between the embryonic development of cardiomyocytes and their differentiation from stem cells (in our case, iPSC), then a paradox arises: simultaneously with an increase in Cx43 expression, the ability to form an electrical connection between cells decreases. This particular contradiction is not resolved by the data of other authors obtained by analyzing the expression of hiPSC-CMs^22^, (Supplementary Fig. 3C). At the same time, expression of Cx43, Cx45, and Cx40 (especially on early differentiation stages) is possible in hiPSC-CMs populations, but the influence of this aspect, as far as the authors know, is still not clarified. The presence of such a paradox serves as indirect evidence in favor of an alternative mechanism for the loss of ability for electrical coupling between cells: for example, an excess of extracellular matrix proteins can break the connection between cardiomyocytes^33^. Thought, is still remains unclear. The role of individual extracellular matrix proteins was identified in preserving the ability to proliferate in cardiomyocytes of newborn rats, for example, recombinant agrin *in vitro* supported division in primary mouse culture cardiomyocytes and cardiomyocytes obtained from human iPSCs^34^. Modulation of signals from integrin receptors can lead to structural changes in the connection of cells, affecting their electrical coupling^35,36^. However, the mechanisms of the transmission of excitation between CMs can vary^17,37–39^ and are not limited to the presence or absence of gap junctions. Instead, we believe that the excitation conduction between cells observed in optical mapping is a sufficient condition for the presence of functional intercellular contacts, when monitoring of intercalated disc formation is very complicated in hiPSC-CMs^12,40^, if special maturation conditions were not applied. Intercellular contacts are a prerequisite for the functional conjugation of an implant and a heart. Therefore, the ability of CMs to form new functional connections plays a key role in modern tissue engineering. Understanding the conditions under which CMs are able to form a sufficient number of new bonds is a crucial point in the development of heart patches based on hiPSC-CMs that functionally bind to a damaged myocardium.

Correct coupling of cardiomyocytes relative to each other is an extremely important factor for the creation of stably conducting tissue. Since modern 3D printing methods cannot fully reproduce the complex structure and specify intercellular connections, one possible solution is the use of self-organization of cardiomyocytes. Cardiomyocytes tend to take the simplest orientation and connectivity without external influences (in this article, cardiomyocytes, seeded before 20 days, reproduced the properties of the control samples, (Fig. 4C). It is likely that the structure of self-organized tissue is the reason for the lower conduction (lower V and C) in the samples transferred after 20 days of differentiation and not showing the ability to organize conducting pathways. Thus, the stable conduction of an excitation wave with a high speed and without discontinuities can be explained by the fact that on the 20th day of differentiation, hiPSC-CMs have the property of self-organization in a conducting tissue that has a sufficient number of pathways. And this ability is dramatically lost after 20 days of differentiation. It is worth noting that the result is in agreement with data on the presence of a critical perinatal window for forming a functional tissue culture when dealing with neonatal rat ventricular myocytes primary cultures.

A more detailed explanation of the process of tissue self-organization and the formation of conducting pathways can be obtained by a detailed CAGE analysis, the time frame of which is proposed in this article: from 12 (the appearance of a stable conduction of an excitation wave) to 20 (a sharp decrease in the ability of cardiomyocytes to electrically couple).

### Limitations

One of the limitations of this work is that during cell transferring we did not sort the cell suspension and did not clear it from the by-products of differentiation. We understand that the cell mass of non-conducting cells can be biologically inert and can significantly affect the attachment and development of CMs^33,37,38,41,42^; however, in our sample comparison, this is compensated for by the constant experimental conditions and, in particular, the fact that the proportion of non-conducting cells in the syncytium remained at the same level (Fig. 2C). Moreover, of fundamental interest here is a rather narrow time range of between 12 and 20 days of differentiation, characterized by a sharp change in the abilities of CMs in the formation of a new functional syncytium.

There are two natural considerations that arise when analyzing the described phenomenon: 1) whether the similar phenomenon can also be seen in other human iPSC lines and 2) whether the differentiation protocol affects the syncytium formation and excitation conduction. To confirm whether the findings in this study depended on cell line or differentiation protocol, we studied another iPSC line (iSMA6L) and that the data analysis confirmed the main hypothesis: the properties of tissue formed by the transplanted hiPSC-CMs depend on a transfer day (data are shown in Supplementary Fig. 2). Also, to a certain extent, an analogy can be drawn with the reliable fact that neonatal rat cardiomyocytes (Worthington protocol) are capable of forming coordinated syncytium when cells are isolated no later than 5 days after rats are born. Speaking about the linking of the observed effect with the differentiation protocol, it is worth noting that the selected (Gi-Wi) protocol ends on the 8th day of differentiation, while the observed effect is manifested in the vicinity of the 20th day of differentiation - 12 days after the end of the protocol. The same is true for most directional differentiation protocols^43^. In this case, it might be more appropriate to associate the observed effect with the conditions under which cardiomyocytes mature.

An important question is whether the efficiency of differentiation in hiPSC-CM varies at different stages of differentiation/maturation, and whether this efficiency can affect the observed effect. Efficiency assessment by the cytofluometry method was carried out only for the 20th day of differentiation, and efficiency monitoring at other stages was carried out on the basis of fluorescence data from optical mapping (see results, Fig. 2D). It should be noted that methods for assessing the effectiveness of differentiation by the total fluorescence recorded with hiPSC-CMs have also been successfully used by other research groups^22^. In our case, the estimate of the number of cardiomyocytes was made by the total fluorescence intensity of Fluo-4AM. There were no significant changes in signal intensity during differentiation *in vitro*. (Supplementary Fig. 1). Therefore, we consider the efficiency of differentiation unchanged throughout the entire period of maturation of the monolayer and equal to the estimated values of cytofluometry performed on the 20th day of differentiation. It worth noting, that cell seeding destiny in transferred samples also was constant (Fig. 2D).

As for the influence of the absolute value of the differentiation efficiency, it makes sense to proceed from the presence of a percolation threshold. Recent studies have shown that the 1: 4 ratio between CMs versus non-CMs is a critical threshold for the formation of the cardiomyocyte conducting system (percolation threshold)^44^. If this threshold is not reached, cardiomyocytes are not able to form the syncytium and maintain the propagation of an excitation wave through the sample. In our case, the ratio is 1:1, therefore, the properties of the formed syncytium are in the zone that is resistant to small changes in the ratio between CMs versus non-CMs^44^.

## Supplementary information

Formation of an electrical coupling between differentiating cardiomyocytes.

## Data Availability

The original experimental data, cell line and plugins for data processing is available from the corresponding author upon reasonable request. The additional information about the cell lines can be found in previous articles^15,16,23^.
