# Supplementary material for: Formation of an electrical coupling between differentiating cardiomyocytes
Source: Sci Rep. 2020 May 8;10:7774. doi: 10.1038/s41598-020-64581-5 (PMC7210299; doi:10.1038/s41598-020-64581-5)
Supplement: Supplementary file 1 — Formation of an electrical coupling between differentiating cardiomyocytes. [file 41598_2020_64581_MOESM1_ESM.pdf]

Formation of an electrical coupling between differentiating cardiomyocytes

M.M. Slotvitsky[1], V.A. Tsvelaya[1], A.D. Podgurskaya[1], K.I. Agladze[1,2]

[1]*Moscow Institute of Physics and Technology, Dolgoprudny, Moscow Region, 141700 Russian Federation*

[2]*M.F. Vladimirsky Moscow Regional Clinical Research Institute, Moscow, 129110 Russian Federation*

Correspondence and requests for materials should be addressed to K.A. (email: agladze@yahoo.com)

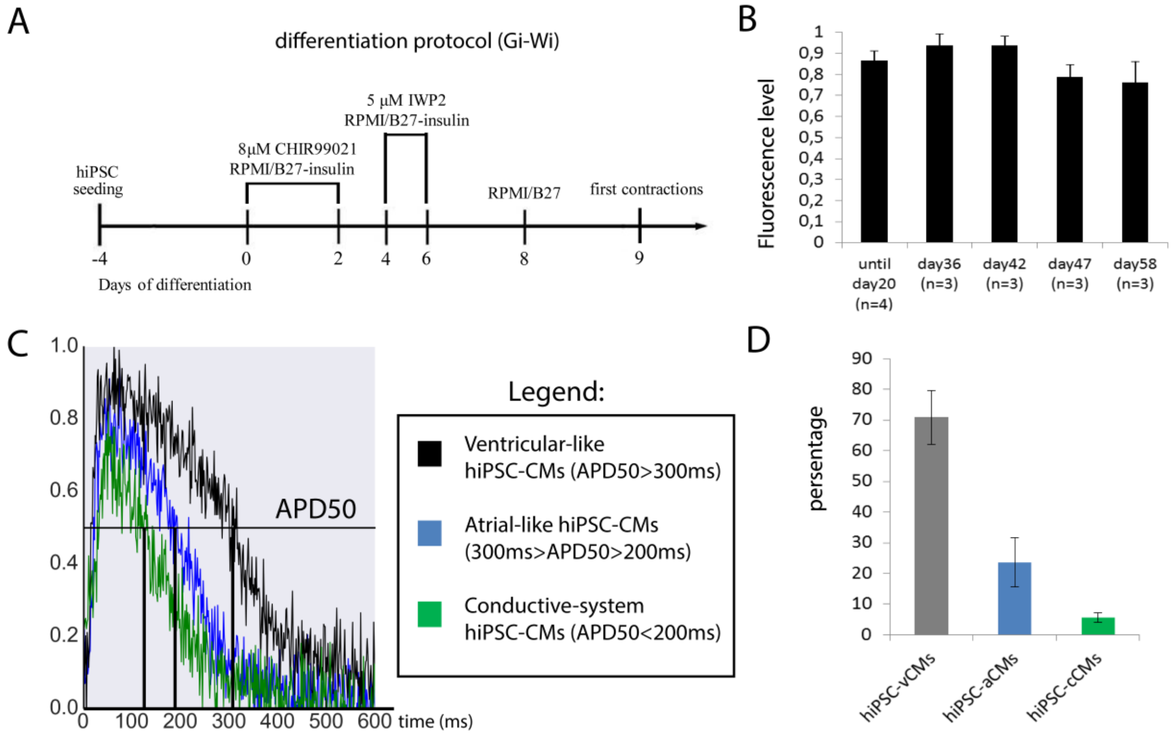

Supplementary Figure 1: Characterization of differentiation efficiency

A. Modification of Gi-Wi protocol for ventricular cardiomyocytes direct differentiation. **B.** Amplitude map (Fluo4-AM) signal intensity during differentiation in vitro. A value of 1 indicates the maximal signal intensity of hiPSC-CMs in differentiated state. There were no significant changes in signal intensity during differentiation in vitro by one-way ANOVA (n=3 each). Values are mean±SD. **C.** Optical recordings of different action potential types (ventricular-like, atrial-like and conductive system) of hiPSC-CMs on 50th day of differentiation. The relative fractions (area) of each action potential type are shown in Figure **D** (n = 5 is the number of different tissue samples).

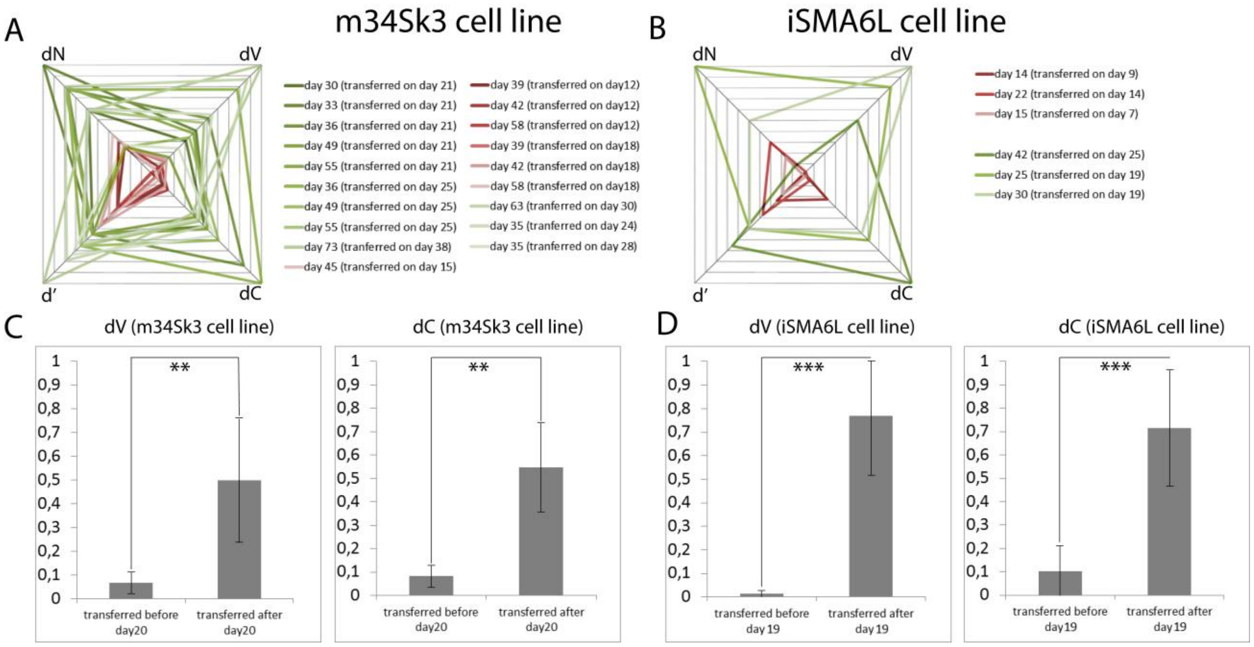

Supplementary Figure 2: Descriptor differences of transferred and control samples

(dN – difference in maximal capture frequency, dV – difference in conduction velocity, dC – difference in correlation value, d' – day of transfer)

A. Petal diagram with descriptors differences for all m34Sk3 transferred samples. A similar diagram for iSMA6L cell line is shown in Figure **B.**

C. Statistical significance of differences in speed (left) and correlation (right) from control values for samples transferred before 20 days or after 20 days of differentiation (m34Sk3 cell line). Values are mean±SD

P-value <0.001 (**) for both by one-way ANOVA (n=7, m=12)

D. Statistical significance of differences in speed (left) and correlation (right) from control values for samples transferred before 19 days or after 19 days of differentiation (iSMA6l cell line). Values are mean±SD. P-value is 0.006 and 0.017 correspondingly for dV and dC by one-way ANOVA (n=3 for each group).

*** p-value<0.05

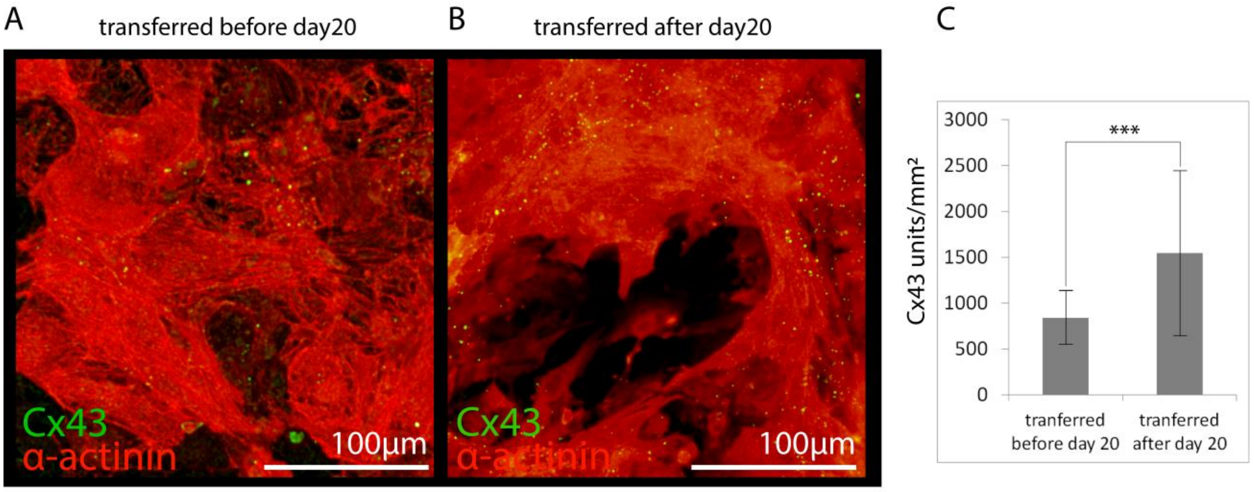

Supplementary Figure 3: Connexin43 distribution in transferred samples (m34Sk3 cell line)

A,B. Immunocytochemistry of different tissue fragments in samples transplanted before the 20th day of differentiation (day19) and after (day27).

C. Comparison of Connexin43 level in samples transplanted before the 20th day of differentiation and after. Values are mean±SD.

*** p-value <0.05 (p= 0.0171 by one-way ANOVA, n=10 in both groups)
